# The Role of Heat Shock Protein 40 in Carcinogenesis and Biology of Colorectal Cancer

**DOI:** 10.2174/1381612828666220513124603

**Published:** 2022-08-01

**Authors:** Fereshteh Asgharzadeh, Reyhaneh Moradi-Marjaneh, Mahdi Moradi Marjaneh

**Affiliations:** 1 Department of Physiology, School of Medicine, Mashhad University of Medical Sciences, Mashhad, Iran;; 2 Department of Physiology, School of Paramedical Sciences, Torbat Heydariyeh University of Medical Sciences, Torbat Heydariyeh, Iran;; 3 Department of Infectious Disease, Faculty of Medicine, Imperial College London, London, United Kingdom

**Keywords:** Heat shock protein 40, chaperone DnaJ, colorectal cancer, carcinogenesis, metastasis, treatment

## Abstract

Colorectal cancer (CRC) is the third most common cancer worldwide. Despite the enormous amount of effort in the diagnosis and treatment of CRC, the overall survival rate of patients remains low. The precise molecular and cellular basis underlying CRC has not been completely understood yet. Over time, new genes and molecular pathways involved in the pathogenesis of the disease are being identified. The accurate discovery of these genes and signaling pathways are important and urgent missions for the next generation of anticancer therapy research. Chaperone DnaJ, also known as Hsp40 (heat shock protein 40), has been of particular interest in CRC pathogenesis, as it is involved in the fundamental cell activities for maintaining cellular homeostasis. Evidence shows that protein family members of DnaJ/Hsp40 play both roles, enhancing and reducing the growth of CRC cells. In the present review, we focus on the current knowledge of the molecular mechanisms responsible for DnaJ/Hsp40 in CRC carcinogenesis and biology.

## 1 INTRODUCTION

Colorectal cancer (CRC) is the third highest in mortality among cancer-related deaths worldwide. Probable causes include genetic factors, environmental factors, and chronic, lingering inflammation. Chemotherapeutic compounds serve as the first line of defense against metastatic CRC. In addition, recently, a combinational treatment with targeted therapy against vascular endothelial growth factor (VEGF), or epidermal growth factor receptor (EGFR), has been proven to improve survival in patients with specific CRC subtypes. Despite increasing advances in identifying the molecular mechanisms of CRC and thus discovering effective therapeutic approaches, metastatic CRC still has a poor survival rate. Hence, discovering novel therapeutic targets is important for developing more effective treatment options and improving the quality of life of patients [1].

**Fig. (1):**
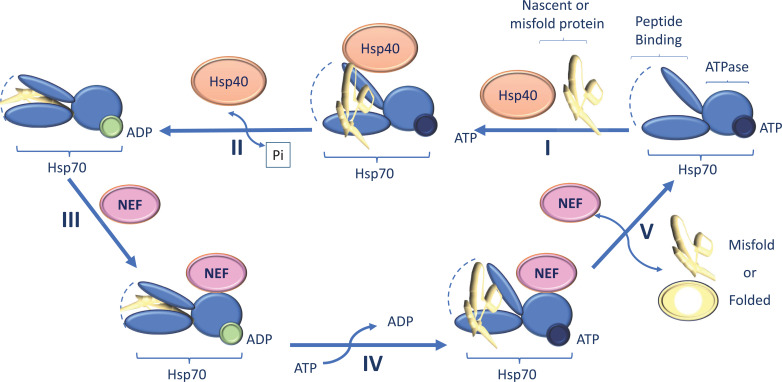
Role of Hsp40 in cancer development.

The heat shock protein 40 (Hsp40), also known as DnaJ, is an understudied family of molecular chaperone proteins. This family is subcategorized into three subclasses because of structural differences: DnaJA (DNAJA), DnaJB (DNAJB), and DnaJC (DNAJC) [2, 3]. The members of this family play a role in protein translation, folding, unfolding, translocation, and degradation, primarily through regulating the activity of Hsp70 chaperon proteins [4, 5]. There is growing evidence revealing the involvement of several isoforms of the DnaJ/Hsp40 family in various types of cancers [6-8]. Protein family members of DnaJ/Hsp40 have both growth-promoting and tumor-suppressing roles (Fig. **1**). Hsp40 has been recently established as a potential target for disease treatment. However, many of the DnaJB proteins to which Hsp40 is attributed have not been completely characterized. Furthermore, the protein product of Hsp40 genes is still under study [9].

Our knowledge about the role of the Hsp40 family in cancer is limited, particularly because of the high number of family members and the diversity between them. Figs. (**2** and **3**) show expression levels of the Hsp40 family members in CRC and their impact on survival outcomes. Hsp40 family members such as Tid I (class DnaJ A3) and HLJ1 (class DnaJB4) have been reported to be involved in the regulation of tumor development [10, 11]. In the present review, we study the published research identifying members of human Hsp40 involved in various aspects of CRC carcinogenesis and biology.

## 2 HSP40 (HEAT SHOCK PROTEIN 40)/CHAPERONE DNAJ

**Fig. (4):**
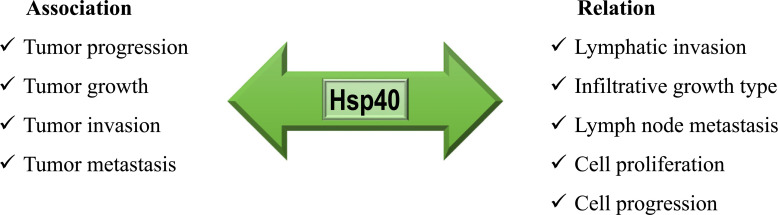
The interaction and regulation of the Hsp70 machinery cycle by Hsp40. I: The delivery of nascent or misfolded substrates to ATP-bound Hsp70; II: Hydrolysis of ATP to ADP; III & IV: NEF (Nucleotide exchange factor) binds to Hsp70, catalyzing the exchange of ADP for ATP; V: ATP binds to the Hsp70’s ATPase domain.

Hsp40 (DnaJ) is an understudied family of chaperone proteins. These chaperons interact synergistically with Hsp70s in extensive biological processes such as protein synthesis, folding, refolding, assembly, translocation, degradation, and regulation of the activity of Hsp70. Despite their importance and diversity, there is still poor information on how they work with Hsp70 chaperone machinery, how they modify the folding characteristics of the bound clients, and how they identify and engage non-native proteins [12]. Hsp70 proteins use the cycle of ATP binding and hydrolysis, coupled with the activity of nucleotide exchange factor (NEF), to regulate its association and dissociation from client proteins. DnaJ/Hsp40s are involved in regulating this cycle through stimulation of the ATPase activity of Hsp70. ATP hydrolysis transforms Hsp70s from an open state with high binding and release rates for substrates to a closed state with low exchange rates and therefore stabilizes Hsp70s interaction with substrates. In addition, they can convert the Hsp70 function by determining client specificity, which probably changes the fate of client proteins (Fig. **4**) [13-16].

DnaJ/Hsp40 family is categorized into three subclasses based on structural differences: DnaJA (4 members), DnaJB (13 members), and DnaJC (32 members). The DnaJA and DnaJB subclasses function independently of ATP and bind to aberrant polypeptides. The DnaJC subclass contains polypeptide binding domains (PPDs) that recognize its specific substrate. Subclass C proteins also rely on an Hsp70 protein partner [2, 3] to function properly. DnaJ proteins bind to unfolded protein substrates by their zinc fingers and C-terminal domains. They also bind to their partner Hsp70s through a very conserved “J” domain, which is usually present in the N-terminal region of the protein. The C-terminal region of Hsp70s also contains a binding site for Hsp40. The “J” domain is essential for stimulating the ATPase activity of Hsp70. In addition to the J domain, there are other conserved regions in DnaJ/Hsp40s proteins are critical to their functions and may characterize their functional diversity [5, 17]. Besides, it has been suggested that DnaJ proteins may possess chaperone-like activity independently of Hsp70 chaperones [18].

The DnaJ/Hsp40 proteins are localized in various intracellular compartments, including the nucleus, cytosol, mitochondria, endoplasmic reticulum, endosome, and ribosomes, and their function depends on their localization. The wide variety of DnaJ/Hsp40 proteins and their localization indicate the numerous actions of these compounds. However, the roles of many of these HSP40 members are not yet well understood [3].

The mechanisms by which DnaJ/Hsp40 proteins interact with Hsp70s are not completely recognized, but briefly, Hsp40 binds to multiple hydrophobic regions in the client protein and delivers to ATP-bound Hsp70 in an unfolded state, which also Hsp70s can conformationally bind [19]. Hsp40 binds to Hsp70 through its J domain and accelerates its ATPase activity. Without any major conformational changes, Hsp70s displace the client from Hsp40 through a simple displacement mechanism. In addition, Hsp40 has a lower affinity for the client which facilitates the transferring process. After hydrolysis of ATP to ADP, Hsp70 undergoes a conformational modification, the closure of the α helical “lid” of the peptide-binding domain, which tightly binds the substrate. Hsp40 also dissociates from Hsp70. Then NEF takes its action and catalyzes the ADP dissociation, which follows displacing ATP from ADP, inducing an “opening” state of the “lid” and releasing the client to fold or pass another chaperone-mediated cycle [12, 20].

## 3 DNAJA1 (HDJ2)

Human protein DnaJ subfamily A member 1 (DnaJA1), also known as Hdj2, is a set of evolutionarily-conserved proteins responsible for various key cellular functions such as protein assembly and disassembly, folding, translocation of proteins into cellular compartments, and degradation [21]. It has also been shown that DnaJA1 regulates androgen receptor signaling and spermatogenesis in mice [22] and facilitates proteins imported into the mitochondria [23]. DnaJA1 function in tumor development is still controversial. In the C6 rat glioblastoma model, DnaJA1 suppresses tumor growth and metastatic formation capacity, which is attributed to diminishing relocation of N-cadherin and the activity of metalloproteinases and prevention of the amoeboid-like transition of tumor cells [24]. DnaJA1 was downregulated 5-fold in a genomic analysis of cancer cells and targeted as a biomarker in pancreatic cancer. In addition, its overexpression diminishes the stress response capabilities of the oncogenic transcription factor, c-Jun, and reduces cell survivability. It is hypothesized that DnaJA1 activates a DnaK protein by making a complex that inhibits the JNK pathway, hyperphosphorylation of c-Jun and antiapoptotic state observed in pancreatic cancer [25]. However, it has been shown that inhibition of DnaJA1 can contribute to radiosensitivity of glioblastoma multiforme cells [26].

In examining the role of DnaJA1 in the development of CRC, Yang *et al.* showed that DnaJA1 was upregulated in tumor tissues and highly associated with serosa invasion, lymphatic metastasis, and poor prognosis. DnaJA1 increased the proliferation, invasion, and metastasis of tumor cells. DnaJA1 was activated by the upstream transcription factor E2F1 and then stimulated the cell cycle and development of CRC through inhibiting division cycle protein 45 (CDC45) ubiquitin degradation. Thus, DnaJA1 was established as a role promoter in CRC. In addition, the function of DnaJA1, including proliferation and metastasis, could be weakened by KNK437. Most importantly, they also revealed that KNK437 combined with Oxaliplatin/5-fluorouracil/leucovorin showed a synergistic inhibitive effect on DnaJA1-mediated liver metastasis, suggesting KNK437 as an alternative approach for the CRC treatment [21].

## 4 DNAJA3 (TID1)

DnaJA3, also known as tumorous imaginal disc 1 (Tid1), belongs to the DnaJ protein family. Like other members of the DnaJ family, Tid1 serves as a cochaperone for binding and activating Hsp70 proteins. It is characterized by a J-domain that binds to Hsp70s to regulate their activity and perform its cochaperone function. Tid1

possesses a long isoform hTid-1(L) and a short isoform hTid-1(S). It has been shown that in response to cytotoxic threats, these isoforms show opposite functions; tid1 (L) and Tid1(S) promote and inhibit apoptosis, respectively [27, 28].

Tid1 is the only mammalian counterpart of the Drosophila tumor suppressor, Tid56, which has been classified as a tumor suppressor [29]. The anticancer activity of Tid1 has been reported in human lung adenocarcinoma cell lines [27], human basal cell carcinoma [30], and glioma cells [31]. Although the anticancer mechanism of Tid1 is not well understood, Kim *et al.* showed that Tid1 negatively regulated the motility and metastasis of breast cancer cells by attenuating the production of interleukin-8 by increasing NF-kb activity on its promoter [10]. Furthermore, Kurzik-Dumke showed that Tid1 might be a negative regulator of ErbB-2 and capable of decreasing the oncogenic signaling mediated by the receptor in breast cancer [32]. In the context of CRC, Tid1 acts as a ligand of the tumor suppressor adenomatous polyposis coli (APC) [33]. In normal colon epithelium, the distribution of these tumor suppressors, Tid1 and APC, is closely related to the noticeable apical-basal polarity in epithelial cells. In CRC, however, Tid1 expression is altered in both aspects of the expression level and cellular distribution. CRC development is associated with overexpression and loss of polarization of expression of the Tid1 tumor suppressor. Evidence shows that in Tid1 and APC’s expression pattern in CRC is associated with a shift from compartmentalized to diffuse cytoplasmic. In addition, the presence of the human Tid1/APC/Hsp70/Hsc70/Axin/Dvl multiprotein complex were confirmed in CRC tissues. These findings suggest that Tid1 function might be critical in the modulating of signaling pathways involved in the regulation of cell polarity and pattern formation. In addition, Tid1 seems to act upstream of APC. Thus, it may change the expression of the latter and affect primarily on downstream members of the wingless/Wnt signaling, a crucial pathway which is hyperactive in CRC [34].

Traicoff *et al.* analyzed the relationship between INT6 and Tid1 and a set of tumor suppressor proteins including Patched, p53, c-Jun, and phosphorylated c-Jun in breast, colon, lung, and ovarian tumor tissues. INT6 is a protein with reduced levels in tumors, and its binding partners are involved in tumorigenesis. They observed mainly significant positive correlations, especially between INT6 and Tid1. These findings suggest that these compounds might play a role in common signaling pathways involved in the cell cycle, growth, and apoptosis [35].

## 5 DNAJB4 (HLJ1)

DnaJB4 (HLJ1) is another member of the DnaJ family of Hsps and is regarded as a tumor suppressor gene in several cancers, including lung, colon, and gastric cancers [36]. HLJ1 plays important roles in proliferation, anchorage-independent growth, motility, invasion, tumorigenesis, and cell cycle progression [11]. However, its biological properties, mechanism of action, and cell signaling pathways affected by HLJ1 are poorly understood. HLJ1 can suppress cancer development and metastasis through complex interacting mechanisms. It has been shown that HLJ1 suppresses the catalytic activity of Src and downregulates the formation of oncogenic complexes related to EGFR, FAK, and STAT3 signaling pathways [37]. In addition, it has been shown that HLJ1 is a caspase-3 substrate and stimulates the sensitivity of cancer cells to apoptosis by increasing JNK and caspase activity [38]. Additional studies are needed to understand the exact mechanism of HLJ1 action.

To detect the implications of HLJ1 in CRC, Liu *et al.* compared HLJ1 expression in different cell lines with different metastasis capabilities. They also compared HLJ1 expression in CRC or lymphatic metastatic tissues with normal mucosa. In addition, the relationship between HLJ1 level and survival rate was examined in their study. They showed that HLJ1 expression was lower in highly metastatic CRC cell lines than in lowly metastatic ones. HLJ1 overexpression significantly inhibited CRC cell proliferation and invasion *in vitro* and was significantly downregulated in CRC or lymphatic metastatic tissues compared with normal mucosa. Furthermore, there was a positive correlation between HLJ1 levels and the survival rate in CRC patients. Liu *et al.* claimed that HLJ1 is a strong tumor suppressor for CRC and could be used as a biomarker to predict the clinical outcome of patients [39].

Growing evidence shows that HLJ1 could be a molecular target in anticancer therapeutics. For example, it has been shown that curcumin, whose anticancer effects have been identified [40-44], targets the tumor suppressor HLJ1 and stimulates its activity and expression [45]. DMSO is also an important stimulator of HLJ1 [46]. In addition, andrographolide, a promising new anticancer herbal agent, can induce HLJ1 expression and suppress growth and invasion in cancer cells [47]. These compounds might be templates for developing novel anticancer drugs that target HLJ1.

## 6 DNAJB6 (MRJ)

DnaJB6, also called a mammalian relative of DnaJ (MRJ), is another Hsp40 family member. There are two splice isoforms of MRJ in humans, a full-length isoform, MRJ(L), and a shorter isoform, MRJ(S). MRJ plays various roles in cell physiological processes, including transcription, cellular signaling, and cell adhesion [48]. It has been reported that DnaJB6 (MRJ) has an important role in controlling and assessing the development and outcome of multiple cancers, although more detailed studies on the role of MRJ in tumor progression are needed [49-52].

Evidence shows an amplification of the MRJ gene in CRC tissues [53] and a positive correlation between increased expression of MRJ and tumor invasion and poor prognosis [8]. Zhang *et al.* showed that, unlike normal tissues, both MRJ(L) and MRJ(S) were overexpressed in CRC [8]. Using CRC cell lines, HCT116 and SW480, they showed MRJ knockdown did not change cell migration but reduced the invasion. MRJ knockdown also reduced CRC lung metastases *in vivo* assays. These data suggest that high levels of MRJ expression increase the invasiveness of tumor cells in human CRC [8]. In another study, Zhang *et al.* observed an upregulation of IQ-domain GTPase-activating protein 1 (IQGAP1) by MRJ through siRNA-mediated silencing [8]. IQGAP1 is a key regulator of cellular functions such as cytoskeletal rearrangements during cell invasion, proliferation, and cytokinesis [54-56]. It acts as a scaffold and binds many proteins, including the oncogene β-catenin, tumor suppressor E-cadherin, and Rho GTPase [57, 58]. An increased expression of IQGAP1 was observed in human CRC, and IQGAP1 silencing inhibited ERK activation [59]. ERK has been shown to increase MMP expression, resulting in degradation of the extracellular matrix and invasion of cancer cells into neighboring tissues [60, 61]. Interestingly, MRJ knockdown inhibited cell invasion but did not affect migration. MRJ is also reported to ease the structuring of the K8/18 filament, which inhibits epithelial cancer cell migration [62]. Therefore, it is claimed that K8/18 may constrain the cell migration induced by IQGAP1 in MRJ-overexpressed CRC cells. In addition, it was shown that MRJ upregulated ERK1/2 and FAK phosphorylation, which suggests that it is involved in the MAPK pathway [63]. Direct interaction of MRJ with intermediate filament keratin 18 (K18) and regulating the structuring of K8/18 have also been reported [9, 64]. uPAR has also been found to interact with K18 and MRJ [65]. These studies collectively suggest MRJ/uPAR interaction may modulate tumor growth.

Several members of the Hsp40 family have been suggested to be key players in tumor progression and metastasis [66-68]. The contribution of MRJ to the pathology of the disease is of great importance. Other than reduced tumor progression in MRJ-expressing cells, they showed decreased secreted metastasis proteins and increased secreted metastasis suppressor levels [50]. It is noteworthy that MRJ is a suppressor of Wnt/β-catenin signaling through multiple pathways. It is believed that Wnt/β-catenin signaling hyperactivation can be the initiating and driving event of CRC. MRJ can stimulate the upregulation of Wnt inhibitor dickkopf 1 (DKK1) and secretary glycoprotein and maintains the dephosphorylation status of glycogen synthase kinase 3β (GSK3β) through the protein phosphatase PP2A. Following these events, the β-catenin is degraded [69]. Hence, MRJ could have a suppressive effect on CRC growth and metastases [48], and one of the molecular mechanisms by which MRJ reverses epithelial-mesenchymal transition (EMT) might be the inhibition of Wnt/β-catenin signaling [70, 71]. With these descriptions, MRJ's function in tumorigenicity, suppression, and metastasis is ambiguous. More research on MRJ function in tumor biology is needed. Further studies are needed to focus on the MRJ-induced signaling and MRJ mutations in tumor progression.

Mitra *et al.* found that miR-632 can negatively regulate MRJ expression [71]. An exogenous miR-632 expression reduced MRJ protein levels and resulted in a population with higher invasiveness. In contrast, silencing endogenous miR-632 reduced the invasiveness of breast cancer cells. Interestingly, an opposite behavior was reported for miR-632 and MRJ expression [72]. The involvement of miR-632 in other cancers regarding MRJ has not been fully characterized yet.

## 7 DNAJB8

Cancer stem‐like cells/cancer‐initiating cells (CSC/CIC) have been considered in cancer treatment. These cells form a small population of cancer cells that express a stem cell phenotype with highly tumorigenic properties. CSCs have been noted for their tumor initiation, self‐renewal, and differentiation ability. It has also been reported that these cells are resistant to chemotherapy and radiotherapy. In addition, they are associated with tumor metastasis and recurrence. Therefore, targeting CSCs in treating tumors, especially their resistant types, is a logical approach [43, 73]. CSCs express several tumor‐associated antigens, which are promising sources for CICs‐targeting immunotherapy. These antigens are not present in normal cells but are abundantly expressed in cancer cells and CSCs. Targeting these antigens could open new windows for novel molecular cancer therapies [74]. Growing evidence shows that DnaJB8, another member of the DnaJ family, is expressed preferentially in the CSCs population of cancer cells among normal tissues [75, 76]. Morita *et al.* identified DnaJB8 as one of the CSC antigens in colon cancer cells and documented its overexpression in CRC [75]. In addition, similar results were reported by Nishizawa *et al.* in renal cell carcinoma [76]. Immunotherapy using DnaJB8 engineered antibodies could be a promising approach to CRC treatment. Fortunately, Tadano *et al.* have recently discovered a single-chain variable-fragment artificial antibody reactive against DnaJB8 [77].

## 8 DNAJC12 (JDP1)

DnaJC12, also named JDP1, is another member of the Hsp40 family, which, like other members of this family, participates in several fundamental biological functions [66]. The human JDP1 gene encodes two isoforms, the full-length JDP1 protein (variant a) and a shorter transcript (variant b). The identical DnaJ domains exist in the N-terminal portions of both isoforms, but the C-terminal portion of variant B is shorter than that of variant a [78]. Although the specific function of JDP1 is yet unknown, the available data show that its level is controlled by physiological stimuli [79]. JDP1 is upregulated in breast tumors [80], and estrogens can stimulate its expression in estrogen-sensitive human breast cancer cells [78]. It is also reported that JDP1 expression levels are related to estrogen and progesterone receptor status and may be valuable in predicting the response to hormonal therapy [81]. To understand the function of JDP1, Choi *et al.* tried to identify the binding partners of JDP1 and revealed that the most frequent partner of JDP1 differed between stressed and unstressed cells. BiP and Hsc70 (cognate Hsp70 chaperone) were frequently associated with JDP1 in stressed and unstressed cells. They also found that JDP1 was upregulated in prostate cancer cells by androgen-induced bZIP CAMP Responsive Element Binding Protein 3 Like 4 (AIbZIP/ CREB3L4). AIbZIP/CREB3L4 is a transcription factor of the bZIP family, which is associated with the membrane of the endoplasmic reticulum (ER). Thus, JDP1 is upregulated by stress condition related to ER [79]. Furthermore, JDP1 is associated with a more aggressive type of gastric cancer. High levels of JDP1 expression are correlated with lymphatic invasion, lymph node metastasis, and progression of gastric cancer. Therefore, high levels of JDP1 indicate a poorer prognosis, suggesting JDP1 as a potential therapeutic target [7]. In the context of rectal cancer, He *et al.* showed overexpression of JDP1 was correlated with poor prognosis for overall survival, disease-free survival, and local recurrence-free survival. High expression of JDP1 also predicted a poor response to chemoradiotherapy in patients with rectal cancer [66].

## CONCLUSION

DnaJ (HSP40) family comprises the largest and most diverse subgroup of Hsps and are major partners for Hsp70 proteins. These proteins regulate extensive biological processes such as protein synthesis, folding, refolding, assembly, translocation, and degradation. These regulatory functions make the DnaJ family more specific and selective drug targets than other Hsps. Although the specific function of many members of this family is unknown, in this article, we provide an overview of the involvement of some members of the human DnaJ/Hsp40 family in various aspects of CRC carcinogenesis and biology. DnaJs are noteworthy in that the members of this family appear to have both activities for and against CRC. Briefly, based on accumulating evidence in this review, certain DnaJ proteins are probably involved in CRC suppression, including DnaJA3 (Tid1) and DnaJB4 (HLJ1), but DnaJB8 and DnaJC12 (JDP1) are likely involved in CRC progression. However, the role of DnaJA1 and DnaJB6 (MRJ) in CRC development is still controversial. Although the mechanism of action of DnaJs is of significant interest, it is yet to be fully explored. Since DnaJs simultaneously affect many biological processes in cells, targeting them in CRC therapy may lead to better outcomes and increase the efficacy of anticancer drugs.

## Figures and Tables

**Fig. (2):**
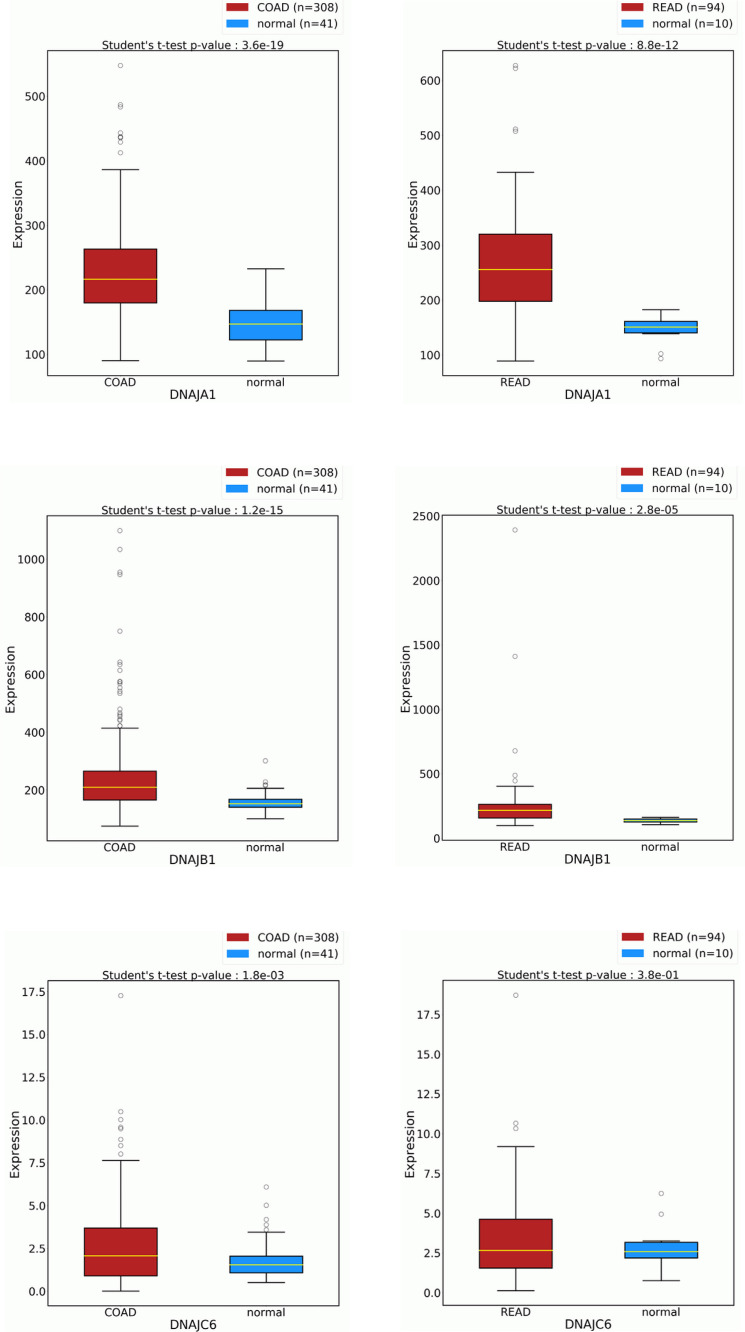
Overexpression of Hsp40 family members in colon and rectum adenocarcinomas (COAD and READ, respectively). In each box plot, gene expression levels are compared between cancer and normal samples. The plots were generated by OncoDB (http://oncodb.org).

**Fig. (3):**
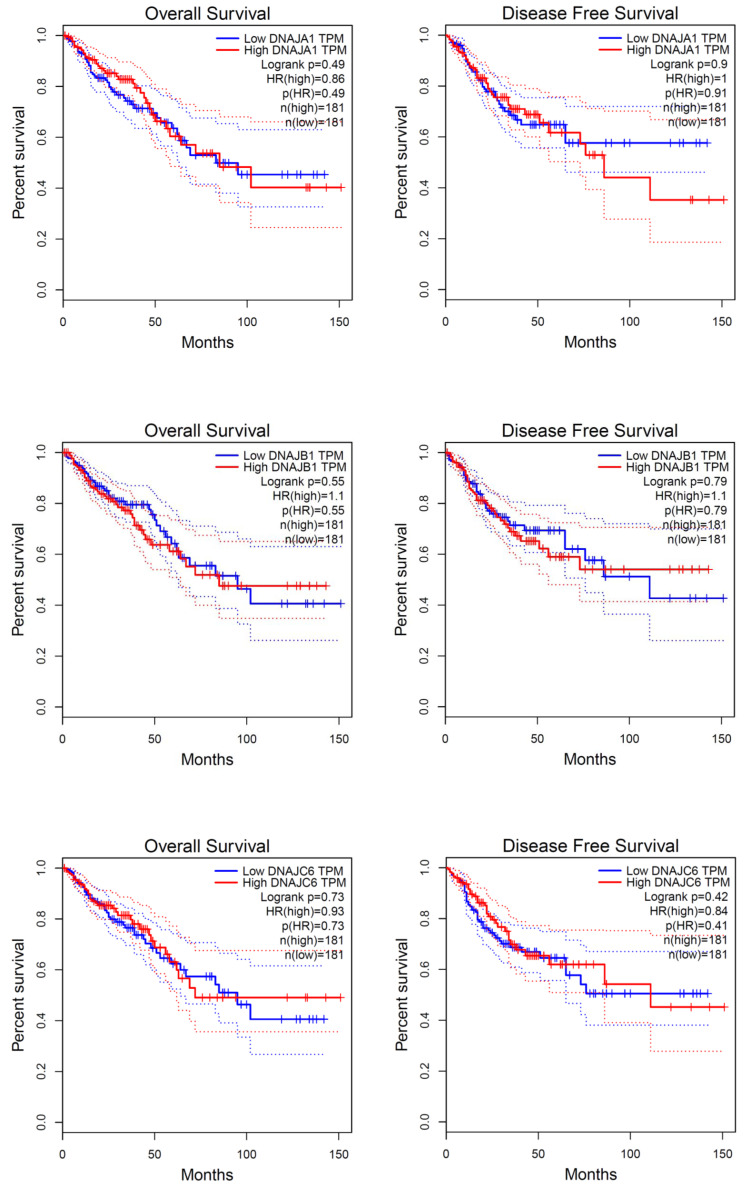
The effect of expression levels of Hsp40 family members on overall survival and disease-free survival. Colon and rectum adenocarcinoma patients were included. TPM and HR represent transcript per million reads and hazard ratio, respectively. The plots were generated by GEPIA (© 2017 Zefang Tang, Chenwei Li, Boxi Kang. Zhang's Lab).

## LIST OF ABBREVIATIONS

APC: Adenomatous Polyposis Coli
CSC: Cancer Stem-Like Cells
CIC: Cancer Initiating Cells
CRC: Colorectal Cancer
NEF: Nucleotide Exchange Factor
VEGF: Vascular Endothelial Growth Factor
